# *S. aureus* virulence factors decrease epithelial barrier function and increase susceptibility to viral infection

**DOI:** 10.1128/spectrum.01684-23

**Published:** 2023-09-22

**Authors:** Mary C. Moran, Matthew G. Brewer, Patrick M. Schlievert, Lisa A. Beck

**Affiliations:** 1 Department of Dermatology, University of Rochester Medical Center, Rochester, New York, USA; 2 Department of Microbiology & Immunology, University of Rochester Medical Center, Rochester, New York, USA; 3 Department of Microbiology & Immunology, University of Iowa, Iowa City, Iowa, USA; Icahn School of Medicine at Mount Sinai, New York, New York, USA

**Keywords:** *Staphylococcus aureus*, atopic dermatitis, keratinocytes, viral infection, superantigens, barrier function, vaccinia virus, USA300, CD40, SElQ

## Abstract

Individuals with atopic dermatitis (AD) are highly colonized by *Staphylococcus aureus* and are more susceptible to severe viral complications. We hypothesized that *S. aureus* secreted virulence factors may alter keratinocyte biology to enhance viral susceptibility through disruption of the skin barrier, impaired keratinocyte differentiation, and/or inflammation. To address this hypothesis, human keratinocytes were exposed to conditioned media from multiple *S. aureus* strains that vary in virulence factor production (USA300, HG003, and RN4220) or select purified virulence factors. We have identified the *S. aureus* enterotoxin-*like* superantigen SE*l*Q, as a virulence factor of interest, since it is highly produced by USA300 and was detected on the skin of 53% of AD subjects (*n* = 72) in a study conducted by our group. Treatment with USA300 conditioned media or purified SE*l*Q resulted in a significant increase in keratinocyte susceptibility to infection with vaccinia virus, and also significantly decreased barrier function. Importantly, we have previously demonstrated that keratinocyte differentiation influences susceptibility to viral infection, and our qPCR observations indicated that USA300 *S. aureus* and SE*l*Q alter differentiation in keratinocytes. CRISPR/Cas9 was used to knock out CD40, a potential enterotoxin receptor on epithelial cells. We found that CD40 expression on keratinocytes was not completely necessary for SE*l*Q-mediated responses, as measured by proinflammatory cytokine expression and barrier function. Together, these findings support that select *S. aureus* virulence factors, particularly SE*l*Q, enhance the susceptibility of epidermal cells to viral infection, which may contribute to the increased cutaneous infections observed in individuals with AD.

**Importance:**

*Staphylococcus aureus* skin colonization and infection are frequently observed in individuals with atopic dermatitis. Many *S. aureus* strains belong to the clonal group USA300, and these strains produce superantigens including the staphylococcal enterotoxin-*like* Q (SE*l*Q). Our studies highlight that SE*l*Q may play a key role by altering keratinocyte differentiation and reducing barrier function; collectively, this may explain the AD-specific enhanced infection risk to cutaneous viruses. It is unclear what receptor mediates SE*l*Q’s effects on keratinocytes. We have shown that one putative surface receptor, CD40, was not critical for its effects on proinflammatory cytokine production or barrier function.

## INTRODUCTION

Atopic dermatitis (AD), also known as eczema, is the most common inflammatory skin disorder, affecting an estimated 230 million people worldwide. AD affects 7% of adults and 15% of children in the United States, with most cases developing in childhood (1 – 3). AD is the first step in the atopic march, so named for the progression from AD to food allergy and allergic airway diseases, including asthma and allergic rhinitis (4). The key features of AD include disruption of the skin barrier and skin dysbiosis characterized by the dominance of *Staphylococcus aureus* (5 – 10). Multiple clinical studies have demonstrated that up to 90% of individuals with AD can be colonized with *S. aureus* and skin colonization strongly correlates with disease severity and measures of barrier dysfunction (11 – 14). Birth cohort studies have shown that infants who develop AD can be identified by early-life skin colonization with *S. aureus*, suggesting that this pathogen may contribute to AD onset (15).

Individuals with AD have a dysfunctional epidermal barrier as shown by increased transepidermal water loss (TEWL), permeability to low molecular weight molecules, and elevated surface pH (less acidic), as well as reduced stratum corneum hydration (6 – 9, 16, 17). Both the stratum corneum and tight junctions, the two key elements of the epidermal barrier, have been shown to be dysfunctional in AD (18, 19). Barrier dysfunction is thought to be the consequence of reduced expression of structural or differentiation proteins, an imbalance of proteases and protease inhibitors, altered lipid composition and structure, genetic mutations, and the itch-scratch cycle (19 – 31). Epidermal barrier disruption is believed to facilitate the increased susceptibility to bacterial and viral infections observed in individuals with AD (32, 33).

Individuals with AD are more susceptible to a number of viral skin complications including eczema herpeticum ([EH], caused by herpes simplex virus [HSV- 1 or −2] infections), eczema molluscatum [caused by molluscum contagiosum virus (MCV) infection), eczema coxsackium (caused by coxsackievirus A16), eczema vaccinatum [caused by vaccinia virus (VV)], and the newly identified eczema monkeypoxicum (caused by mpox) (3, 34, 35). These observations indicate that the epithelium of AD individuals is unable to effectively limit cutaneous viral infections. A potential contributor to this occurrence is skin colonization by *S. aureus*. In support of this hypothesis, a previous study observed that 78% of individuals with AD with a history of EH reported a history of *S. aureus* infections, compared to only 29% of individuals with AD without a history of EH (36). These observations led us to hypothesize that *S. aureus* colonizing the skin enhances epidermal viral susceptibility.

*S. aureus* produces a number of secreted virulence factors that adversely affect host immune responses and tissues. These virulence factors include superantigens, cytotoxins, proteases, and lipases, among others (37). *S. aureus* proteases have been implicated in skin barrier disruption by degrading the barrier proteins desmoglein-1 and filaggrin. In addition, *S. aureus* skin infection models in mice have demonstrated altered cellular localization of tight junction proteins (38, 39). Other studies have found that the *S. aureus* cytolysin, α-toxin, increases the viral entry of HSV-1 or VV (40). Associations between toxic shock syndrome toxin-1 (TSST-1) and EH have also been reported (41). Our study focuses on select superantigens, as virtually all AD *S. aureus* isolates produce superantigens, and AD severity strongly correlates with superantigen abundance (11, 42, 43). At least 26 unique *S. aureus* superantigens have been identified, and these include TSST-1, staphylococcal enterotoxin family (SEs; *N* = 11), and staphylococcal enterotoxin-*like* family (SE*l; N* = 14) (44). Our results identified important *S. aureus*-secreted virulence factors that significantly impact keratinocyte cellular biology and function.

This study utilized conditioned media from three *S*. *aureus* strains USA300 (FRP3757; a derivative of the LAC strain), HG003 and RN4220 (both derivatives of the NCTC8325 isolate—National Collection of Type Cultures 8325, London, UK) (45 – 47). These were selected due to their differences in the production of secreted virulence factors. The original NCTC8325 strain and many subcultures of the organism have been used for years to study virulence properties. Genome sequencing revealed two regulatory mutations in NCTC832, *rsbU* and *tcaR. rsbU* is an activator of SigB, and the mutation in *rsbU* has been shown to reduce virulence, while the mutation in *tcaR* appears to have little measurable effect on virulence. Strain RN4220 is a heavily mutagenized variant of NCTC8325 to render the RN4220 restrictionless, and thus highly useful for cloning genes into *S. aureus*. Strain HG003 is a recently constructed variant of NCTC8325 in which both the *rsbU* and *tcaR* mutations have been repaired (46). Neither RN4220 nor HG003 secretes superantigens, although both have the gene for SE*l*X (45), and neither strain secretes large amounts of cytotoxins, although it is reported that HG003 produces less cytotoxin than the parent 8325 strain (46). RN4220 and HG003 are related to USA400 (multilocus sequence type 1) strains.

*S. aureus* LAC (USA300; multilocus sequence type 8) was first isolated in Los Angeles County. The organism is a community-associated methicillin-resistant strain associated with significant skin and soft tissue infections, and cases of necrotizing pneumonia (48). *S. aureus* LAC produces the superantigens SE*l*K, SE*l*Q, and SE*l*X. The organism is also highly hemolytic and positive for the production of α-toxin (cytotoxin) and Panton-Valentine leucocidin (PVL), as well as selected other cytotoxins. USA300 is also one of the dominant community-associated methicillin-resistant *S. aureus* strains that is associated with AD skin colonization (49, 50). USA300 belongs to the clonal complex eight lineage, which is one of the common lineages associated with AD and disease severity (51, 52).

Using conditioned media from these three strains, as well as purified virulence factors, we have identified SE*l*Q as a virulence factor of interest, as it was commonly detected on the skin of individuals with AD, altered keratinocyte differentiation, decreased tight junction barrier function, and increased susceptibility to viral infection.

## RESULTS

**Fig 1:**
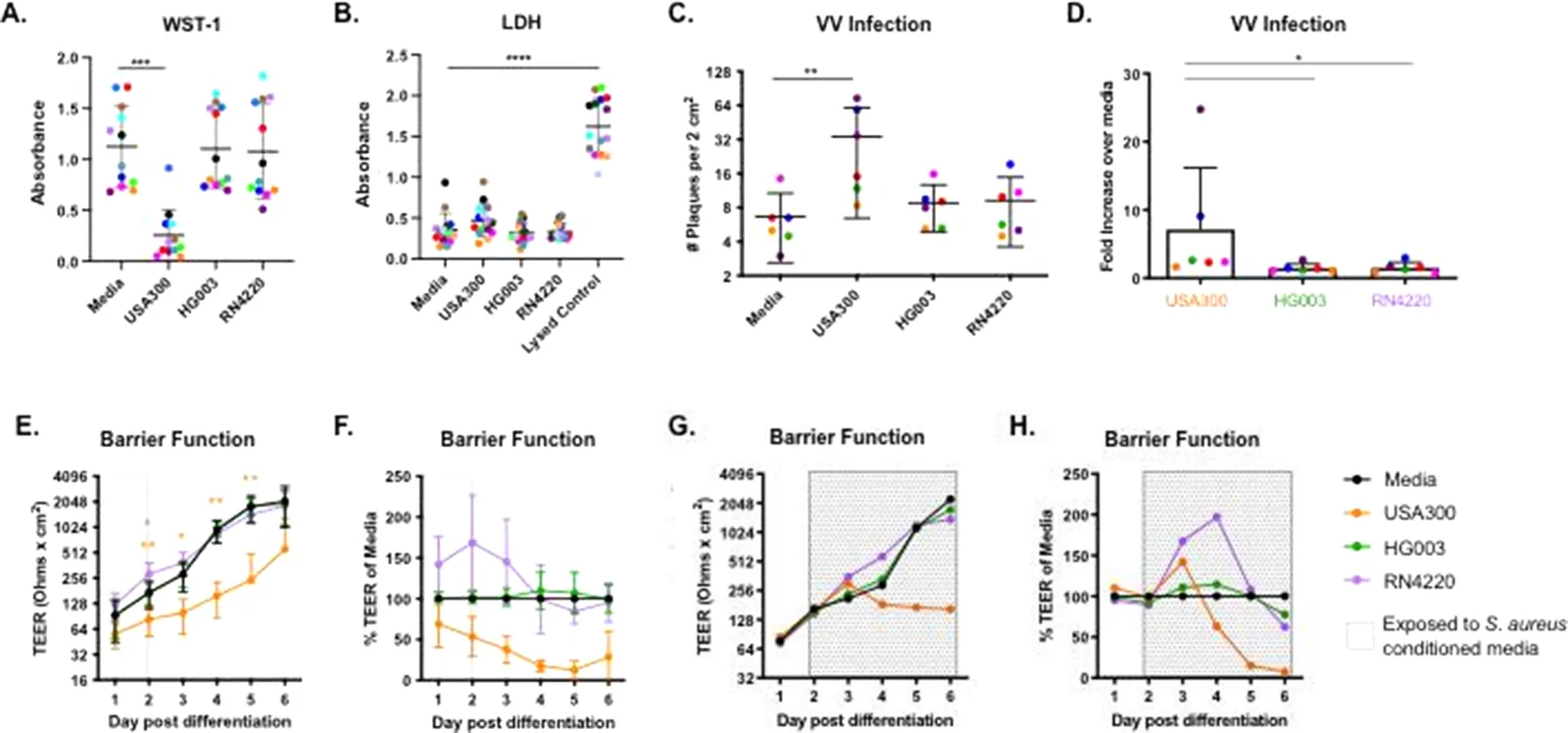
Secreted factors from USA300 significantly reduce keratinocyte metabolism, increase viral susceptibility, and decrease barrier function. PHFK were treated with 15 µg/mL of conditioned media from three *S*. *aureus* strains (USA300, HG003, and RN4220) at the time of differentiation. Two days after treatment, cells were analyzed by WST-1 (**A**) or LDH (**B**), *n* = 14 PHFK. PHFK were infected with a low multiplicity of infection (0.0001) of VV 2 d post-treatment/differentiation. Infection was quantified using crystal violet staining to determine plaque number (**C**) and this was also expressed as fold change over media (**D**), *n* = 7 PHFK. Transepithelial electrical resistance (TEER) was measured for 6 d following differentiation, shown as adjusted TEER values (ohms × cm^2^) (**E**) or percent TEER of media alone (**F**), *n* = 7 PHFK. Differentiation was induced in PHFK and cells were treated with 15 µg/mL of conditioned media 2 d post-differentiation, TEER was measured daily (**G and H**), *n* = 2 PHFK. Each color represents a different PHFK donor (**A-C**). Error bars denote mean ± standard deviation. Significance was calculated by a nonparametric paired ANOVA with Dunn’s multiple comparison test (**A-D**) or mixed-effects ANOVA with Dunnett’s multiple comparisons test (**E**). *P < 0.05, **P < 0.01, ***P < 0.001, ****P < 0.0001

Primary human foreskin keratinocytes (PHFK) were treated with conditioned media from three *S*. *aureus* strains: USA300, HG003, and RN4220 (45 – 47). *S. aureus* conditioned media were used because live bacteria quickly overgrow in epidermal culture media and lead to keratinocyte death. Conditioned media also enabled us to specifically test the impact of secreted virulence factors rather than affects from host cell-to-bacterial contact. Treatment of PHFK with USA300 conditioned media resulted in significant decreases in cellular metabolism compared to media treatment alone, as measured by the WST-1 assay (Fig. 1A). Treatment with HG003 and RN4220 conditioned media had no impact on cellular metabolism. The lactate dehydrogenase (LDH) assay, used to assess cellular integrity, showed no difference between *S. aureus* conditioned media and media alone (Fig. 1B). Together, these assays indicate that PHFK treated with USA300 conditioned media have altered cellular metabolism but are not undergoing cell death.

Since individuals with AD are highly colonized with *S. aureus* and are more susceptible to viral skin complications, we hypothesized that treatment of PHFK with *S. aureus* conditioned media would increase viral susceptibility. PHFK were treated with 15 µg/mL (total protein content) of USA300, HG003, and RN4220 conditioned media at the time of differentiation. Two days later, cells were infected with a low multiplicity of infection (MOI) of VV. Cells were infected at 2 d post-differentiation, since at this timepoint, cells are less susceptible to infection, and therefore we would detect increases in infection upon treatment (53 – 55). Treatment of PHFK with the USA300 conditioned media resulted in a significant increase in VV plaque number, while HG003 and RN4220 conditioned media resulted in no change compared to media treatment (Fig. 1C and D).

We next tested whether treatment with *S. aureus* conditioned media would impact tight junction barrier function, as this could be an explanation for the increased susceptibility to viral infection (20, 40, 53). PHFK were grown in transwells and treated with 15 µg/mL of USA300, HG003, and RN4220 conditioned media at the time of differentiation. Cells were exposed to the conditioned media for the first 2 d of differentiation, and then wells were replaced with fresh media (without *S. aureus* conditioned media treatment). Treatment with USA300 conditioned media for just the first 2 d of differentiation resulted in significant decreases in barrier function at days 2–5 of differentiation as measured by transepithelial electrical resistance (TEER) when compared to media alone (Fig. 1E and F). This indicates that exposure to USA300 conditioned media during the first 2 d of differentiation resulted in lasting changes to epidermal barrier function. We also observed a transient enhancement in barrier function in cells treated with RN4420 conditioned media, while treatment with HG003 conditioned media had no effect (Fig. 1E and F). We also tested how exposure to the *S. aureus* conditioned media later in the process of differentiation would impact tight junction barrier formation and function, modeling what might occur when *S. aureus* is present in the more superficial layers of the epidermis. Exposure to USA300 conditioned media starting at day 2 of differentiation prevented the continued development of barrier as observed through a plateau in TEER (i.e., TEER did not increase over time once treated), while the RN4220 conditioned media again caused a transient increase in TEER (Fig. 1G and H). These results indicate that the complex mixture of secreted factors from pathogenic *S. aureus* (USA300) significantly impairs tight junction barrier formation in keratinocytes, compared to relatively nonpathogenic (i.e., low superantigen producing) *S. aureus* strains.

**Fig 2:**
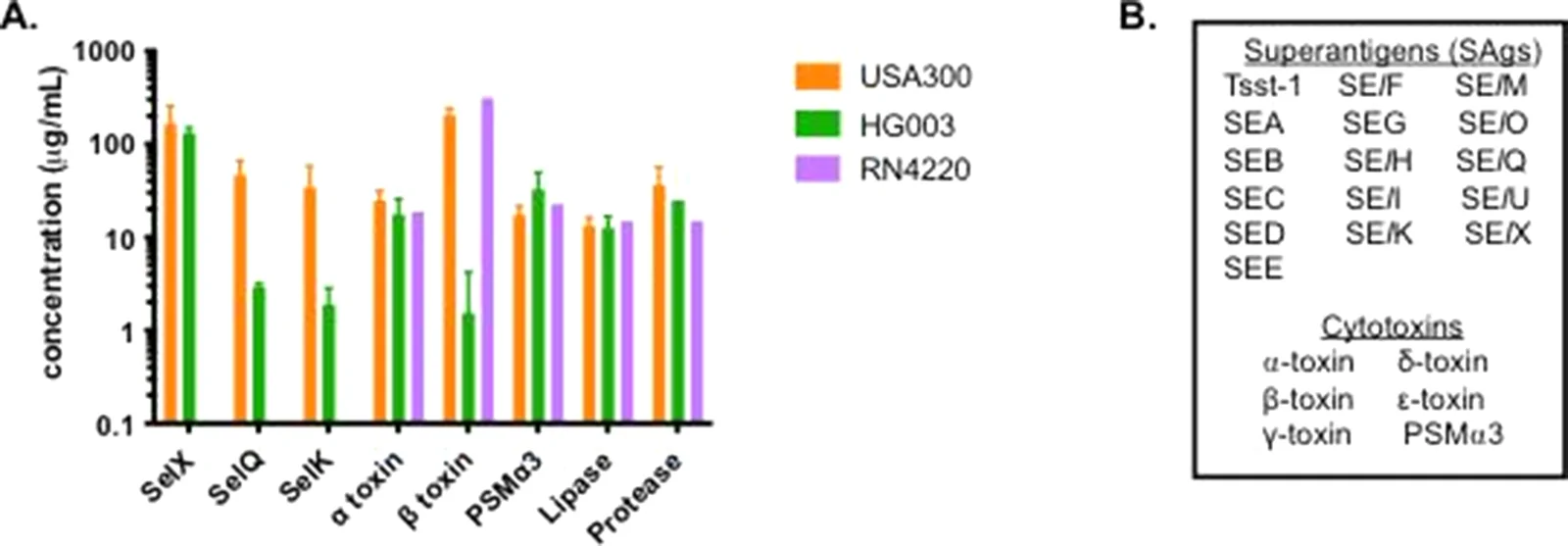
Identification of SE*l*Q as a virulence factor of interest. (**A**). *S. aureus* virulence factors were measured in conditioned media from USA300, HG003, and RN4220 as described in references (56, 56). (**B**). List of superantigens (staphylococcal enterotoxins (SE) and staphylococcal enterotoxin-*like* toxins (SE*l*), enterotoxins and toxic shock syndrome toxin-1 (TSST-1)) and cytotoxins measured

Using the methodology outlined in Moran et al. (56), the secreted virulence factors present within the USA300, HG003, and RN4220 conditioned media were quantified. The secreted virulence factor profiles among these strains were consistent with what is in the literature (37, 45 – 47). The greatest difference in virulence factor production across the three strains was in the production of two *S*. *aureus* SE*l* toxins, SE*l*Q and SE*l*K. These were highly produced by USA300, with minimal to no secretion by HG003 (background/limit of detection), and were not detectable in RN4220 (Fig. 2A). Among AD subjects in a large cohort, SE*l*Q was detected on 53% of AD subjects (*n* = 72) at either lesional or nonlesional sites (manuscript under review). Due to the high production of SE*l*Q by the USA300 strain, and its detection on the skin of AD subjects, the effects of SE*l*Q on keratinocytes were investigated in more detail.

**Fig 3:**
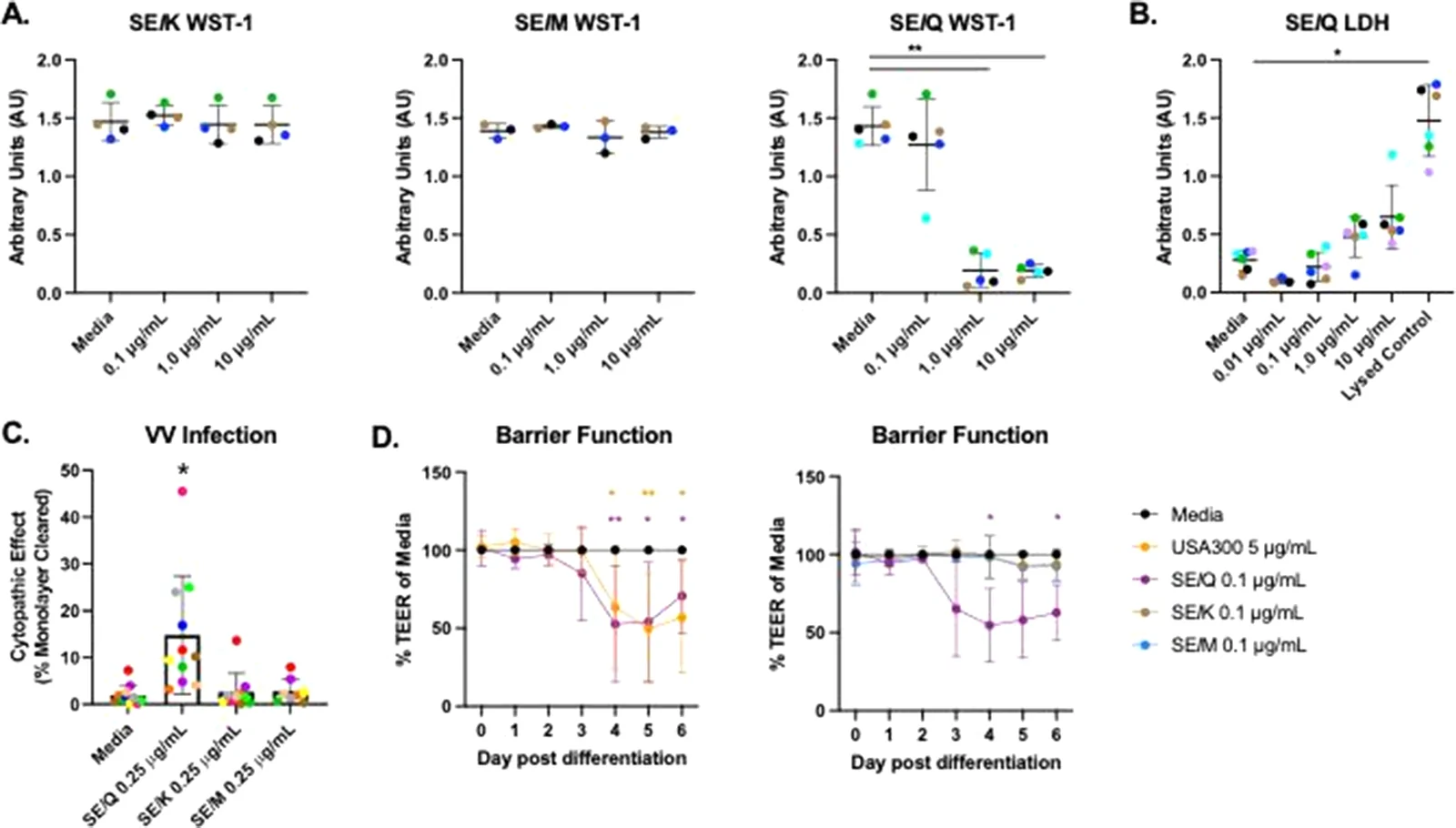
SE*l*Q decreases keratinocyte metabolism, increases viral susceptibility, and decreases tight junction barrier function in differentiated keratinocytes. PHFK were treated with SE*l*Q, SE*l*K, and SE*l*M at the time of differentiation. Cells were analyzed with the WST-1 (**A**) or LDH (**B**) assays 2 d post-treatment, *n* = 3–6 PHFK. (**C**). PHFK were treated with SE*l*Q, SE*l*K, and SE*l*M at the time of differentiation and infected with a MOI 0.0001 of VV 2 d later. Infection was quantified using crystal violet staining to determine the percentage of each well in which VV plaques cleared the monolayer (cytopathic effect), *n* = 11 PHFK. (**D**). PHFK were treated with USA300 conditioned media or purified toxins (SE*l*Q, SE*l*K, and SE*l*M) at the time of differentiation. TEER was measured for 6 d following differentiation; *n* = 4–8 PHFK. Error bars denote mean ± standard deviation Significance was calculated by a nonparametric paired ANOVA with Dunn’s multiple comparison test (**A**), the nonparametric unpaired ANOVA with Dunn’s multiple comparison test (**B**) (comparing each treatment to the media control), and the mixed-effects ANOVA with Dunnett’s multiple comparisons test (**C and D**). Statistics were calculated on Ohms × cm^2^ values (**D**). Each color represents a different PHFK donor (**A, B**). *P < 0.05, **P < 0.01, ***P < 0.001

PHFK were treated with purified SE*l*Q to test whether this superantigen was the predominant driver within the USA300 conditioned media of altered cellular metabolism, increased susceptibility to viral infection, and decreased barrier function. PHFK were also treated with purified SE*l*K or SE*l*M, as these are the two SE*l* toxins most homologous to SE*l*Q (57). SE*l*K was also more highly produced by USA300 than HG003 or RN4220 (Fig. 2A). Doses ranging from 0.1 to 10 µg/mL were tested. Superantigens can be present in biofilms at 15,000 µg/mL, and the *S. aureus* toxin TSST-1 has been measured in blister fluid from patients with bullous pemphigoid, an autoimmune blistering disease, in concentrations as high as 19.4 µg/mL, suggesting that the doses we utilized are of physiological relevance in the skin (58, 59). Treatment of PHFK with purified SE*l*K or SE*l*M had no impact on cellular metabolism as measured by the WST-1 assay, while treatment with purified SE*l*Q (one or 10 µg/mL) resulted in significant decreases in cellular metabolism (Fig. 3A). Similar to treatment with the USA300 conditioned media, treatment with purified SE*l*Q did not result in significant changes in cell membrane integrity as measured by the LDH assay (Fig. 3B). These findings indicate that the doses of purified SE*l* toxins used in our subsequent assays (0.25 and 0.1 µg/mL) were not causing cell death.

PHFK were next treated with 0.25 µg/mL of purified SE*l*Q, SE*l*K, or SE*l*M. This dose was equivalent to the concentration present within the USA300 conditioned media which was utilized in Fig. 1. This concentration is also within the range that virulence factors had been measured on the skin of patients with AD in Moran et al. 2019, with concentrations ranging from 0.01 to 29.5 µg/mL (56). Treatment with purified SE*l*Q resulted in significantly increased susceptibility to VV infection at day 2 post-differentiation, while treatment with SE*l*K or SE*l*M had no effect (Fig. 3C). To test the effect of SE*l*Q on tight junction barrier function, PHFK were treated with 5 µg/mL (protein amount) of USA300 conditioned media or a dose-matched 0.1 µg/mL of purified SE*l*Q. Both of these treatments resulted in significant reductions in TEER at days 4–6 of differentiation (Fig. 3D). As was observed in the cellular metabolism and viral infection assays, treatment with purified SE*l*K or SE*l*M also had no significant impact on TEER (Fig. 3D). Overall, these results are in accordance with our results from USA300 treatment, suggesting that SE*l*Q is an important secreted virulence factor in these responses and phenotypes.

**Fig 4:**
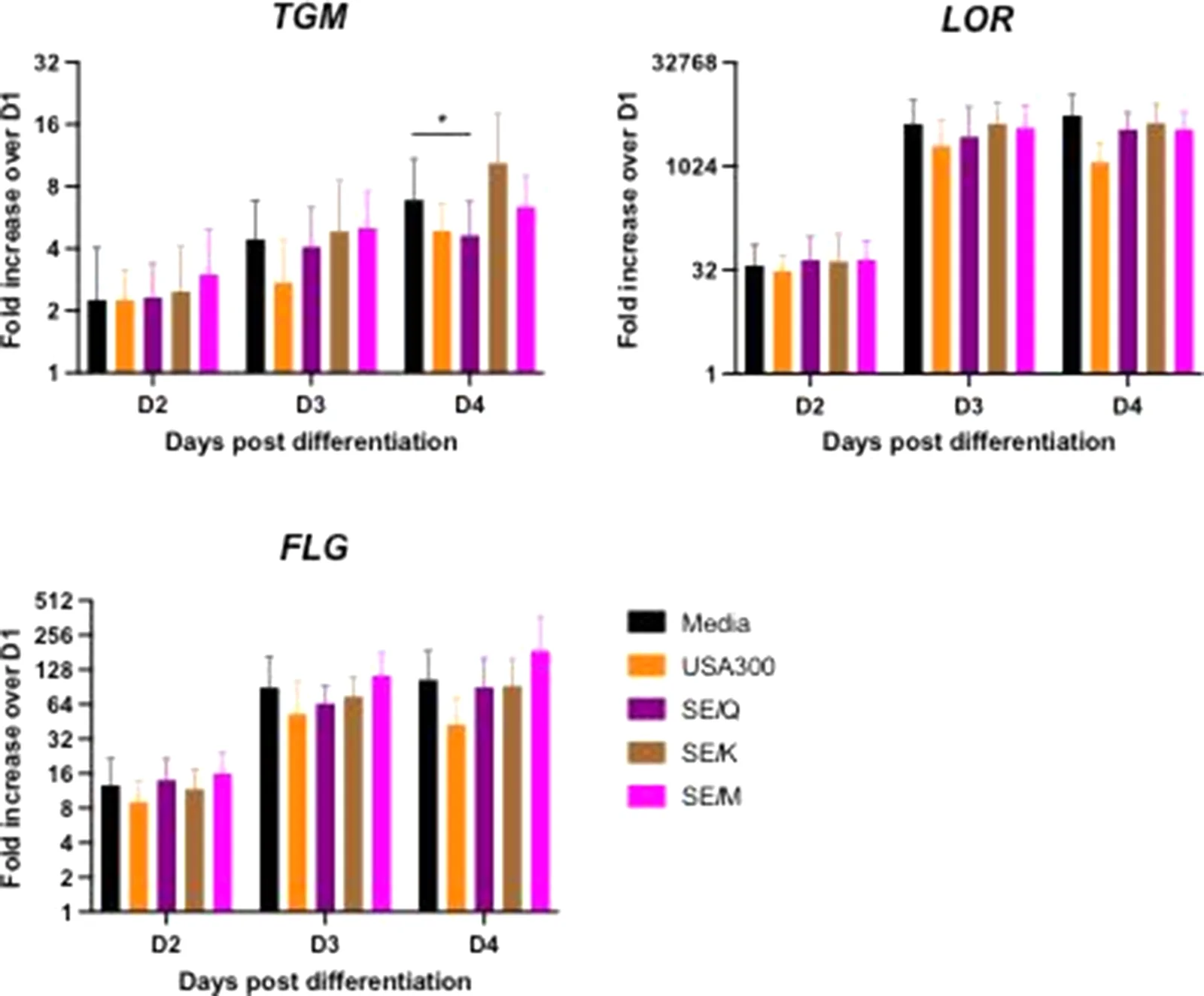
USA300 and SE*l*Q decrease mRNA expression of markers of keratinocyte differentiation. PHFK were treated with 0.1 µg/mL of *S. aureus* toxins (SE*l*Q, SE*l*K, and SE*l*M) or 5 µg/mL of the USA300 supernatant at the time of differentiation. Cell lysates were collected at days 1–4 post-differentiation (**D1-D4**). mRNA expression of the differentiation markers filaggrin (FLG), loricrin (LOR), and transglutaminase-1 (TGM-1) was determined by qPCR. Data are expressed as the fold increase in expression compared to the D1 levels (log 2 scale). Error bars denote mean ± standard deviation. Significance was calculated on the delta cq values using the mixed-effects ANOVA with Dunnett’s multiple comparisons test. *P < 0.05; *n* = 6 PHFK.

We have previously demonstrated that the stage of keratinocyte differentiation significantly influences susceptibility to viral infection (54, 55). It is also known that the process of keratinocyte differentiation is necessary for the development of tight junction and stratum corneum barrier function. We hypothesized that one explanation for the enhanced viral susceptibility and decreased barrier function observed following treatment with USA300 conditioned media or purified SE*l*Q could be that these treatments were altering the normal keratinocyte differentiation process. Therefore, PHFK were treated with USA300 conditioned media, purified SE*l*Q, SE*l*K, or SE*l*M at the time of differentiation, and cell lysates were collected at days 1–4 of differentiation. Quantitative PCR (qPCR) was used to measure the relative expression of mRNA transcripts for markers of keratinocyte differentiation, including filaggrin (FLG), loricrin (LOR), and transglutaminase-1 (TGM-1) (60). While we did not observe many significant differences in expression (fold increase in expression over day 1 levels, log2 scale) across our treatment groups, transcripts of all differentiation markers (TGM-1, LOR, and FLG) showed the same trend in diminished expression in USA300 compared to media-treated cells (Fig. 4). Decreases with SE*l*Q treatment were also observed, and there were significant differences in TGM-1 expression over time in SE*l*Q-treated cells (Fig. 4). While reproducible trends were observed, there was substantial variability in the data which is common when using PHFK. Differentiation kinetics can vary across donors making it difficult to discern differences in this assay and others (which we have previously reported on) (54).

Figures 1–4 demonstrated that treatment with USA300 conditioned media caused significant changes in keratinocyte biology and function, identified SE*l*Q as a virulence factor of interest, and showed that treatment with purified SE*l*Q had comparable effects to those observed with the USA300 conditioned media. Our next aim was to understand how SE*l*Q was exerting these effects on keratinocytes. A previous study demonstrated that the toxins TSST-1 and SEB utilize the receptor CD40 to induce proinflammatory cytokine production in human vaginal epithelial cells (61). We therefore investigated whether SE*l* toxins required CD40 to trigger proinflammatory responses from keratinocytes by creating CD40 knockout (KO) cells utilizing CRISPR/Cas9. The N/TERT-2G cell line was used to generate CD40 KO cells, as these cells are more suitable than PHFK for genetic manipulation due to the ability to propagate these cells for numerous passages. We have previously shown N/TERT-2G to be comparable to PHFK, including in responses to *S. aureus* conditioned media (54).

**Fig 5:**
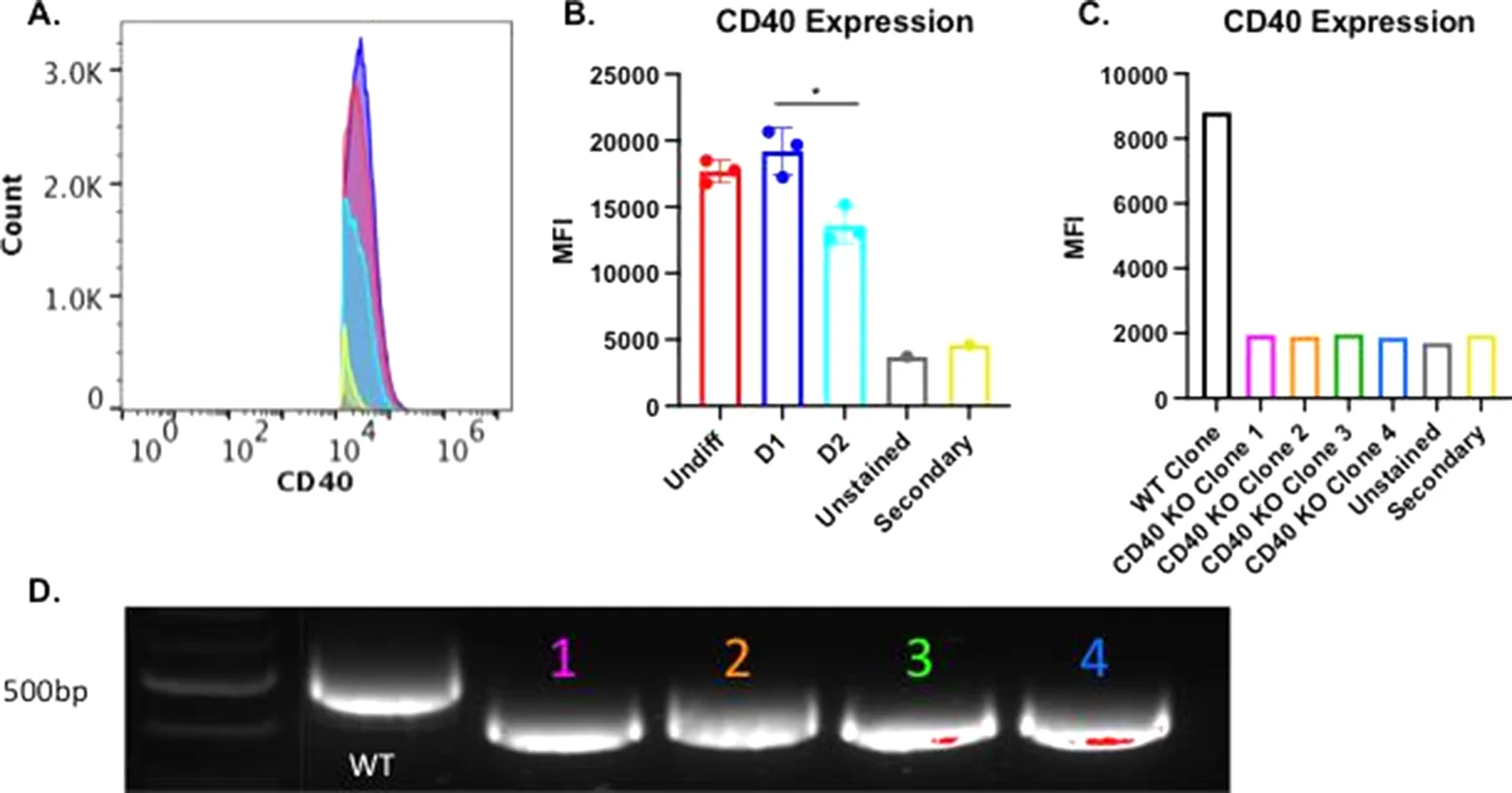
Establishment of CD40 KO in N/TERT-2G keratinocytes. (**A and B**) Undifferentiated (Undiff) and differentiated keratinocytes at day 1 and 2 post-differentiation (**D1, D2**) were stained for CD40 expression and analyzed by flow cytometry. The fourth exon of CD40 was targeted through triple gRNA-mediated CRISPR/Cas9. The clonal selection was utilized to isolate individual clones. (**C**) Undifferentiated CD40 KO clones (1–4) and the WT clone were stained for CD40 expression and analyzed by flow cytometry. (**D**). KO was confirmed by gel electrophoresis for CD40 deletions and by sequencing. Statistics were calculated using the nonparametric unpaired ANOVA with Dunn’s multiple comparison test. Error bars denote mean ± standard deviation. *P < 0.05

CD40 expression on N/TERT-2G cells was confirmed by flow cytometry, which demonstrated a decrease in CD40 expression over the course of differentiation (Fig. 5A and B). Upon confirming CD40 expression on keratinocytes, CRISPR/Cas9 was used to knock out CD40 in N/TERT-2G cells. The fourth exon of CD40 (upstream of the transmembrane region) was targeted through a triple guide RNA-mediated CRISPR/Cas9 reaction. The clonal selection was used to isolate five clones from our polyclonal CD40 KO population. CD40 KO was confirmed by flow cytometry for CD40-stained cells, in which CD40 was detected on the surface of a WT clone but not detected on any of the CD40 KO clones (Fig. 5C). Gel electrophoresis further demonstrated that one of our clones had retained CD40 (CD40 WT clone) while four CD40 KO clones had been isolated (Fig. 5D). Sanger sequencing confirmed a deletion in exon 4 of CD40 in all four CD40 KO clones.

**Fig 6:**
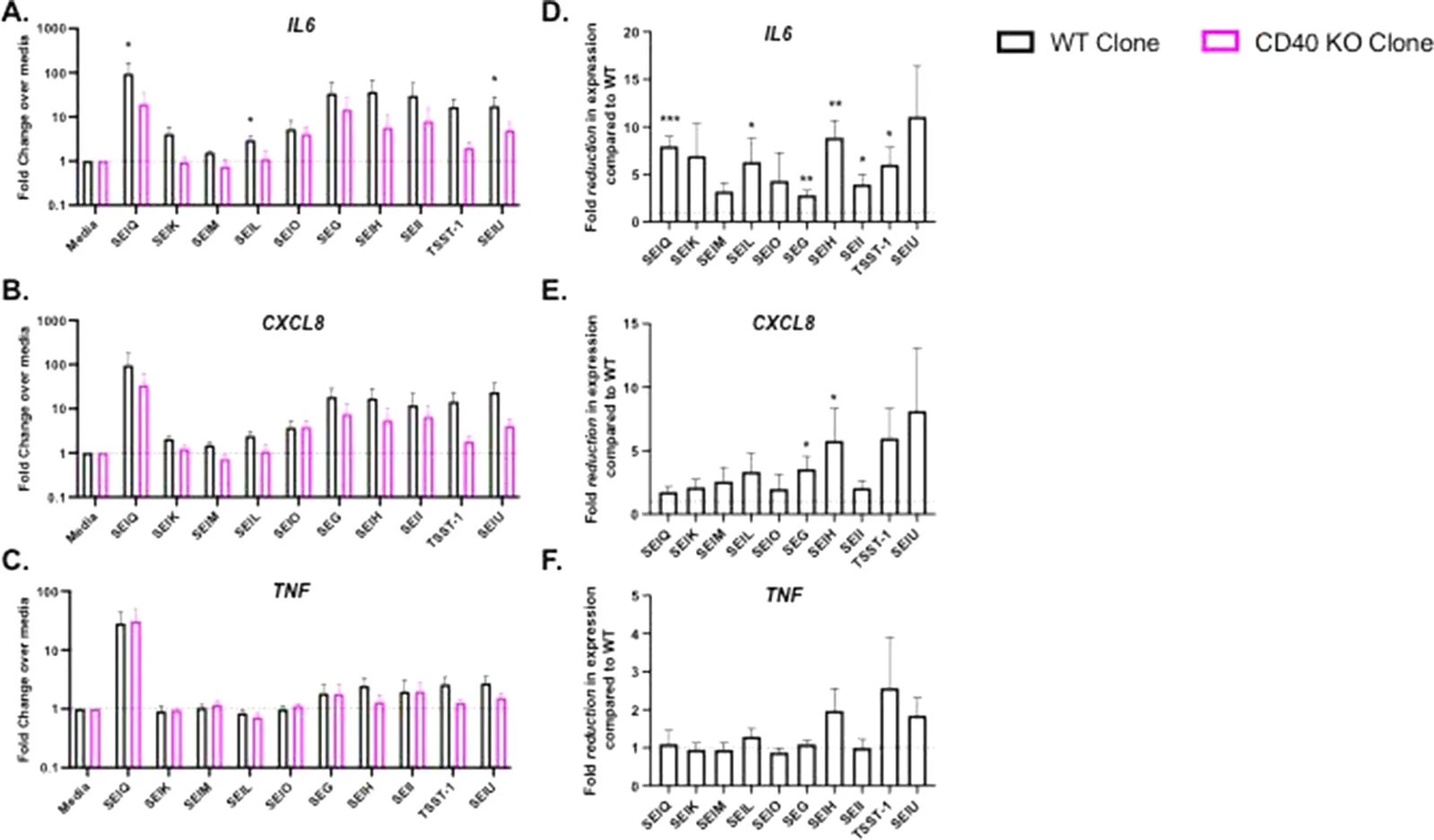
CD40 KO decreases proinflammatory responses to *S. aureus* enterotoxins/SE*l* toxins. The WT clone and CD40 KO clone 1 were treated with purified *S. aureus* toxins (0.25 µg/mL) for 6 h while undifferentiated. Proinflammatory cytokine mRNA expression was determined by qPCR and displayed as “fold change over media” for both WT clone and CD40 KO clone. Statistical analysis was performed using the mixed-effects ANOVA model with Dunnett’s multiple comparisons test comparing the delta cq values of each toxin treatment to media within each clone population (**A-C**). “Fold reduction in expression compared to WT” was calculated by the difference in fold change over media for CD40 KO clone 1 compared to fold change over media for the WT clone. Statistical analysis was performed using the ratio paired *t*-test on the delta cq values comparing WT and CD40 KO (**D-F**). Error bars denote mean ± standard error the mean. *n* = 5 experiments *P < 0.05, **P < 0.01, ***P < 0.001.

After confirmation of CD40 KO clones, we tested how keratinocytes responded to stimulation with purified virulence factors. The WT clone and CD40 KO clone 1 were treated with 0.25 µg/mL of purified toxins (SE*l*Q, SE*l*K, SE*l*M, SE*l*L, SE*l*O, SEG, SE*l*H, SE*l*I, TSST-1, and SE*l*U) for 6 h while undifferentiated, and cell lysates were collected for RNA isolation, cDNA synthesis, and qPCR. Transcripts for *CXCL8*, *IL6,* and *TNF* were measured, as these have been shown to be downstream of CD40 ligation in keratinocytes (62 – 64). We observed that the SE*l* toxins SE*l*Q, SE*l*L, and SE*l*U significantly increased mRNA expression of *IL6* compared to media-treated cells in the WT clone (Fig. 6A). While we observed reproducible trends of increased mRNA expression of *CXCL8* and *TNF* in WT cells treated with SE*l*Q, this did not reach significance (Fig. 6B and C). None of the toxins significantly increased the expression of *IL6, CXCL8,* or *TNF* in the CD40 KO clone 1 (Fig. 6A through C). When comparing the expression levels of *IL6* and *CXCL8* in WT and CD40 KO cells, we observed a significant difference in expression between the cell lines when stimulated with SE*l*Q, SE*l*L, SE*l*H, SE*l*I, and TSST-1 (Fig. 6D and E). These findings indicate that some toxins are able to induce proinflammatory cytokine mRNA expression in WT cells, and that this stimulation can be significantly decreased, but not fully lost, in CD40 KO cells.

**Fig 7:**
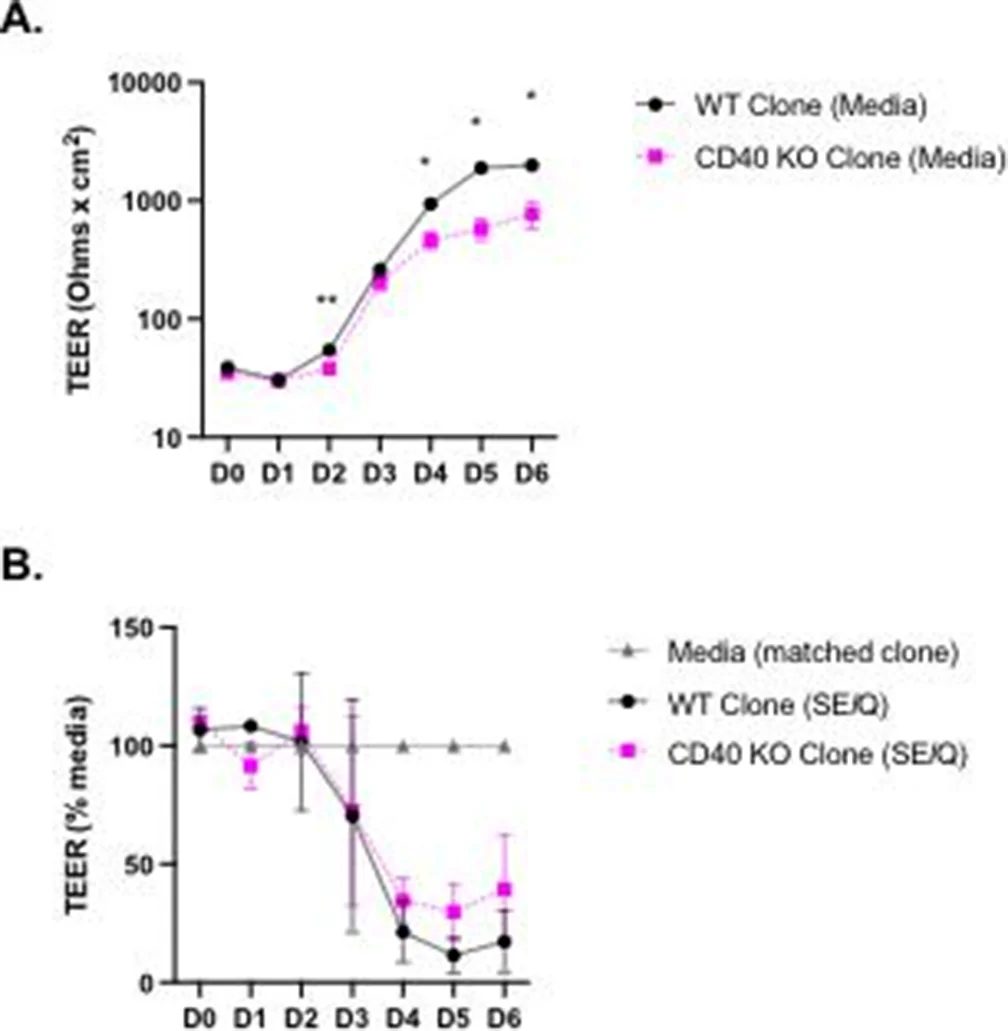
CD40 KO decreases basal tight junction barrier function but does not prevent the barrier disruption caused by SE*l*Q. The WT clone and CD40 KO clone 1 were treated with high Ca^2+^ media [1.8 mM] to initiate differentiation and treated with media alone (**A**) or media with 0.25 µg/mL of SE*l*Q (**B**). TEER was measured for 6 d following differentiation, and media with treatment groups was resupplied every 2 d. Data are shown as adjusted TEER values (ohms × cm^2^) (**A**) or percent TEER of media (matched clone) (**B**). Ratio paired *t*-test, *n* = 3 (**A**). Paired *t*-test, *n* = 3 (**B**). Error bars denote mean ± standard error the mean. *P < 0.05, **P < 0.01

While CD40 expression was not solely necessary for proinflammatory responses, we tested whether CD40 expression was necessary for SE*l*Q to reduce tight junction barrier function. We observed a significant reduction in TEER in untreated (standard Dulbecco's Modified Eagle Medium [DMEM] supplemented with 1.8 mM calcium) CD40 KO cells compared to the WT cells (Fig. 7A). Despite this baseline difference in TEER between the WT and CD40 KO cells, when treated with purified SE*l*Q, this superantigen was still able to further reduce tight junction barrier function in CD40 KO cells (Fig. 7B). When normalizing the data to the respective untreated (media) WT or CD40 KO clone, there was no difference in the percent TEER of media between the WT and CD40 KO clones treated with SE*l*Q (Fig. 7B). These findings indicate that CD40 expression is not required for SE*l*Q to alter barrier formation in keratinocytes.

## DISCUSSION

Keratinocytes are in constant contact with the microbiome colonizing the skin. Bacteria, and specifically *S. aureus*, produce numerous secreted factors that aid in colonization, infection, and immune evasion. We sought to explore the impact of *S. aureus* virulence factors, with a specific interest in SE*l* toxins, on keratinocyte biology with a focus on key characteristics of AD skin including differentiation, barrier function, and susceptibility to viral infection. We hypothesized that *S. aureus* SE*l* toxins alter keratinocyte biology to enhance viral susceptibility through disruption of the skin barrier, impaired keratinocyte differentiation, and/or inflammation.

Treatment of keratinocytes with USA300 conditioned media, which contained a higher concentration of SE*l* toxins than the relatively nonpathogenic strains (HG003 and RN4220), as well as treatment with the purified SE*l* toxin, SE*l*Q, resulted in increased susceptibility to infection with VV. One hypothesis to explain this increased susceptibility to viral infection could be because these *S. aureus* products decrease epithelial barrier function. This has been shown in HSV-1 infection of keratinocytes, in which silencing of the tight junction barrier protein claudin-1 resulted in decreased barrier function (lowered TEER and increased permeability) and an increase in HSV-1 infection (20). We have also demonstrated that keratinocyte tight junction barrier function can be disrupted by treatment with a peptide with extensive amino acid homology to the extracellular loop of claudin-1 (65). Treatment of keratinocytes with this peptide resulted in decreased TEER, altered expression and/or localization of tight junction-related proteins (claudin-1 and occludin), and increased susceptibility to infection with VV (53, 65). We observed that treatment with USA300 conditioned media or purified SE*l*Q also decreased tight junction barrier function (TEER). In this assay, keratinocytes were exposed to these treatments at the time of differentiation, modeling the situation where basal epidermal cells are being exposed to *S. aureus* virulence factors. It is important to note that *S. aureus* has been detected as deep as the dermis (66, 67). Similar effects on tight junction barrier formation and function were observed when USA300 conditioned media was added at d 2 of differentiation, which may be a closer model of *S. aureus* interaction with superficial layers of the epidermis. Other studies have demonstrated a biphasic effect of *S. aureus* (strains SH1000 and 6820) colonization on keratinocyte barrier function, in which short-term (<12 h) incubation resulted in increased tight junction barrier function, while long-term (>24 h) colonization resulted in decreased barrier function. These effects were shown to be due to changes in the localization of barrier proteins and *S. aureus*-induced proinflammatory cytokines (68). Overall, these alterations in tight junction barrier function may contribute to the increased susceptibility to infection we observed. Changes in epithelial barrier function may result in enhanced access to viral entry receptors or greater access to cells more susceptible to infection. It has been shown that treatment with the *S. aureus* α-toxin, which can decrease the epithelial barrier, also increases viral (HSV-1 and VV) entry and load (40).

Keratinocytes undergo a complex process of differentiation during which there are significant changes in gene and protein expression and localization (69). This process of differentiation is necessary for the formation of a fully functional skin barrier. We have recently demonstrated that the stage of keratinocyte differentiation influences susceptibility to viral infection (54, 55). This may be due in part to the differences in barrier function observed in keratinocytes during the process of differentiation; however, changes to barrier function alone cannot fully account for these differences in susceptibility. For example, undifferentiated keratinocytes, which have no tight junction barrier function, are highly resistant to infection, spread, and the cytopathic effects of VV (54, 55). This indicates that there are gene and protein expression changes outside of epithelial barrier genes/proteins that contribute to the differences in susceptibility to viral infection. This could be the result of changes in expression, localization or access to viral entry receptors, or changes in the expression of antiviral proteins. The transcription factor specificity protein-1 (Sp1) has been shown to be decreased in AD subjects with a history of the viral complication EH, and silencing of *Sp1* in cell culture resulted in enhanced VV infection (70). Data suggest that Sp1 activity changes with differentiation and could therefore contribute to the changes in viral susceptibility we observed in keratinocytes at different stages of differentiation (71, 72). Determining whether Sp1 expression, localization, and activity change with USA300 or SE*l*Q will be the focus of future studies. The antimicrobial peptide cathelicidin has also been shown to have antiviral effects, likely by damaging the viral envelope (73, 74). Cathelicidin expression is also decreased in acute and chronic AD lesions (75). Differential expression of differentiation, antiviral, and antimicrobial proteins could contribute to viral susceptibility observed at different stages of keratinocyte differentiation.

We hypothesized that treatment of keratinocytes with USA300 conditioned media or purified SE*l*Q may result in an alteration of the normal differentiation process, extending the length of time that keratinocytes are highly susceptible to viral infection. Expression of FLG, TGM-1, and LOR should increase as keratinocytes differentiate, and we displayed the data as the fold increase in expression compared to the day 1 values. The magnitude of these changes in expression over the course of differentiation was lower in cells treated with USA300 conditioned media, and some decreases were observed in SE*l*Q-treated cells compared to untreated cells. Expression of FLG, LOR, and TGM-1 have been shown to be decreased in AD skin and AD animal models (60). Changes in differentiation as indicated by decreases in gene expression of these markers may contribute to the decreased barrier function and increased susceptibility to viral infection we observed.

The impact of *S. aureus* superantigens including enterotoxins, SE*l* toxins and TSST-1 on keratinocytes is not well studied. In addition to determining whether SE*l* toxins altered epithelial barrier function, cellular metabolism, and susceptibility to viral infection, we aimed to test the necessity for one of the proposed receptors. The surface molecule CD40 has been shown to be required for toxins (TSST-1, SEB) to trigger a proinflammatory cytokine response from human vaginal epithelial cells (61). CRISPR/Cas9 knockout of CD40 on keratinocytes resulted in decreased proinflammatory cytokine responses, as measured by mRNA expression (qPCR), but not a total loss of response. These observations suggest that while these toxins may be utilizing CD40 in part to induce proinflammatory cytokine responses, CD40 expression is not solely necessary for this response and there is likely a redundancy in receptors. The differences in the reduced responsiveness between WT and CD40 KO cells after treatment with the different toxins also suggest that specific toxins may utilize CD40 for signaling to a greater degree than other toxins. Another hypothesized surface receptor is the surface glycoprotein 130 (gp130), and it was demonstrated that the superantigen staphylococcal enterotoxin A (SEA) can bind and signal through gp130 on adipocytes (76). It is possible that some superantigens could be utilizing gp130 or other receptors on keratinocytes to induce signaling responses in the absence of CD40 expression. Since we have demonstrated that it is possible to use CRIPSR/Cas9 to edit genes in N/TERT-2G cells, future studies could use this methodology to test the necessity of other hypothesized surface receptors.

CD40 ligation and signaling have been shown to play roles in keratinocyte proliferation and differentiation (62, 63, 77). CD40 activation by the CD40 ligand was shown to reduce keratinocyte proliferation and induce differentiation (63). This may be why, in the absence of CD40 (CD40 KO cells), tight junction barrier function was decreased. Without the expression of CD40, these keratinocytes may be missing a signal to shift from proliferation to differentiation and barrier formation. This is supported by our data showing that in the presence of CD40 ligand, proliferation was significantly decreased in the WT cells compared to the CD40 KO cells. Despite this baseline defect in keratinocyte barrier function, we observed that treatment with SE*l*Q was still able to decrease tight junction barrier function in CD40 KO cells. These findings support that CD40 expression is not solely required for SE or SE*l* toxins to alter barrier formation, but it is important in the robust development of skin barrier.

We identified SE*l*Q as a virulence factor of interest due to the high prevalence on AD subjects (56) (manuscript under review), the greater concentration in USA300 conditioned media, which was the conditioned media with the greatest effects on keratinocyte biology, and due to the significant responses in keratinocytes treated with purified SE*l*Q. SE*l*Q can be found in about half of AD subjects, but viral complications from HSV or molluscum contagiosum virus are only seen in 7–10% of AD subjects. This disparity is likely due to the fact that while our *in vitro* studies demonstrate increased susceptibility to infection after SE*l*Q exposure, many other factors *in vivo* are at play that may counteract this enhanced susceptibility. Our group has also demonstrated that type 2 cytokines (IL-4 and IL-13) increase susceptibility to viral infection, further highlighting the complex interactions between the microbiome, host immune responses, and viral infection in AD (53).

There is very little known about how SE*l*Q interacts with keratinocytes or its potential role in AD and it is not a well-studied virulence factor. There has been an increase in the number of studies investigating *S. aureus* virulence factors on the skin in the context of AD, but due to the large number of *S. aureus* virulence factors, few studies have fully explored the impact of SE and SE*l* toxins. A 2016 paper analyzed the capacity of *S. aureus* strains isolated from AD patients to produce different virulence factors, with a focus on SE and SE*l* toxins (78). When comparing strains collected from AD patients in 2008, to strains collected from AD patients between 2011 and 2014, it was observed that the prevalence of virulence factors shifted over time. For example, in the 2008 cohort, the *sel-q* gene was detected in 40% of the AD isolates; however in the 2011–2014 cohort, this gene was only detected in 12% of isolates. There were also significant differences in virulence factor production by race. However, even more recently the percentage of cystic fibrosis *S. aureus* clinical isolates with *sel-q* was 33–40% (79). Other studies have also noted differences in *S. aureus* strain colonization and virulence factor production based on geographic location or environment (80). All of these factors complicate studying these virulence factors in the clinical setting. Furthermore, the field is somewhat limited by a lack of commercially available reagents such as antibodies against these virulence factors, which makes assay development challenging.

Historically, superantigens have been referred to as pyrogenic toxin superantigens to recognize them as being among the most potent pyrogens known (81, 82). However, the large family of pyrogenic toxin superantigens has multiple shared but many unique biological properties. Pyrogenic toxin superantigens have relatively shared three-dimensional structures, with at least 13 amino acids in the same position in space (83), with the rest of the structure folded around those amino acid residues to form each unique serotype. These superantigens are divided into five subfamilies, groups I–V, based on differences in structures that lead to differences in biological activities (81, 82). SE*l*K and Q, as studied in this manuscript, belong to the understudied group V superantigen subfamily (81, 82). This means these two pyrogenic toxin superantigens are pyrogenic, interact with the β-chains of T lymphocyte receptors (but with unique binding patterns) and α and β-chains of MHC II molecules, lack emetic activity, have a unique 15 amino acid sequence that modifies the activity of the molecules as superantigens (81, 82), but variably bind to one or more epithelial cell receptors. The current studies suggest that even within the group V superantigen subfamily, important differences may occur with interaction with keratinocyte receptors. This likely accounts for the greater activity of SE*l*Q than SE*l*K in effects on keratinocytes.

In conclusion, we have demonstrated that *S. aureus* secreted virulence factors, and specifically purified SE*l*Q, significantly alter the biology and function of human keratinocytes. Together, these changes result in increased susceptibility to viral infection. Our findings add further evidence that the interplay between the host microbiome and skin can be an important factor that drives viral susceptibility, which is most notable in AD.

## MATERIALS AND METHODS

### Cell cultures

PHFK were isolated from discarded human neonatal foreskin tissue. Patient consent for experiments was not required because human tissue left over from surgery was de-identified and considered discarded material. The use of de-identified and discarded human skin tissues for research use was approved by the Research Subject Review Board at the University of Rochester Medical Center (URMC IRB STUDY: 00004672). Isolation and propagation procedures for PHFK were done as previously described (84). N/TERT-2G cells were provided by Ellen H. van den Bogaard (Department of Dermatology, Radboud Institute for Molecular Life Sciences, Radboud University Medical Center, 6500 HB, Nijmegen, The Netherlands) and grown as previously described (85, 86). PHFK and N/TERT-2G were switched to DMEM media supplemented with 1.8 mM Ca^2+^ and 4 mM glutamine to induce differentiation. Days post-differentiation refer to the number of days since exposure to the supplemented DMEM.

### *S. aureus* culturing and quantification

The *S. aureus* strains USA300 (FRP3757), HG003 and RN4220 (the latter two being NCTC8325 derivatives) were grown overnight with shaking at 37°C in tryptic soy broth. Cultures were then centrifuged at 7000× *g* for 10 min and filtered through 0.2 µm filters twice to remove bacteria and establish what is referred to as “*S. aureus* conditioned media” throughout this paper. Protein content was determined using the Pierce BCA Protein Assay Kit. The virulence factors present in the *S. aureus* conditioned media were measured using the quantitative Western dot blot assay described in Moran et al. (56).

### WST-1/LDH

PHFK and N/TERT-2G were plated at a density of 75,000 cells/well in 96-well plates. Cells were treated with *S. aureus* conditioned media or purified SE*l* toxins at the time of differentiation. Cell culture media and a lysed cell control were collected 48 h later for use in the Cytotoxicity Detection Kit^PLUS^ (LDH assay - Roche). The LDH assay was incubated for 10 min and then read at 490 nm/620 nm wavelength. A 1:10 dilution of WST-1 (Cell Proliferation Reagent WST-1, Roche) was added to the cell culture wells (final dilution of 1:20 for WST-1 reagent) 48 h after treatment and incubated at 37°C for 2 h after the addition of the reagent. The WST-1 assay was read at 420 nm/620 nm.

### VV infection assay

PHFK and N/TERT-2G were plated at a density of 150,000 cells/well in a 24-well plate. Cells were infected with a low multiplicity of infection of the Western Reserve strain of VV (MOI 0.0001) at 2 d post-differentiation and treatment with *S. aureus* conditioned media or SE*l* toxins (Day 2). Crystal violet was added to the cells 48–72 h after the initial infection. ImageJ software was used to calculate the percentage of the monolayer within each well that was cleared by plaques (cytopathic effects). To do this, each well was selected with the region of interest tool (circle), and the image was duplicated (right click, duplicate). The total area of the circle was determined using Analyze - > Measure. Next, the outside of the circle was cleared using the Edit - > Clear Outside command then the Threshold function was applied to the image so that the cleared monolayer (plaques) was white: Image - > Adjust - > Threshold - > Apply. Finally, all areas considered to be plaques were selected using the Edit - > Selection - > Create Selection, and the selection was inverted using the Make Inverse function. The area covered by plaques was measured as above (Analyze - > Measure), and the area covered in plaques was divided by the total area to obtain the percent monolayer cleared.

### Transepithelial electrical resistance

TEER measurements were done as previously published (20). Cells were plated in transwells (6.5 mm insert, 0.4 µm polyester membrane, Costar). Measurements of TEER were collected for up to 6 d after the initiation of differentiation and exposure to treatment groups (*S. aureus* conditioned media, *S. aureus* SE*l* toxins).

### qPCR

PHFK were plated in 24-well plates, grown to confluency, and treated with *S. aureus* conditioned media or purified SE*l* toxins at the time of differentiation for 1–4 days. CD40 KO or WT clones (N/TERT-2G) were treated with purified SE*l* toxins for 6 h while undifferentiated. To isolate mRNA, culture media was removed, and 250 µL of TRI Reagent Solution (Thermo Fisher Scientific) was added to each well for 5 min. Wells were scraped with P1000 micropipette tips and transferred to Eppendorf tubes. mRNA was isolated from cells using the E.Z.N.A Total RNA Kit (Omega Bio-Tek), mRNA resuspended in nuclease free water, and then quantified by nanodrop. cDNA synthesis was performed with 300 ng of RNA per reaction using the qScript cDNA synthesis kit (Quantabio). The PCR amplification protocol for cDNA synthesis was as follows: 22°C for 5 min, 42°C for 30 min, and 85°C for 5 min. Samples were then prepared for qPCR using 5 µL PerfeCTa SYBR Green SuperMix (Quantabio), 3.6 µL nuclease free water, 1 µL primer and 0.4 µL cDNA, and then run on an iCycler iQ Real-Time PCR Detection System (Bio-Rad) using the following protocol: 94°C for 3 min, 39 cycles 94°C for 15 s and 55°C for 1 min, 95°C for 1 min, 55°C for 55 s and 55°C for 5 s. Primer sequences for each gene transcript investigated are provided in the Table 1 below. RPLP0 was used as the housekeeping gene for Fig. 4 since this remains stable over the course of differentiation**,** while HPRT was used as the housekeeping gene for Fig. 6. Data are shown as the fold change in 2^^-ΔCq^ values either over the day one matched treatment (Fig. 4) or the fold change in 2^^-ΔCq^ values over the media control (Fig. 6).

**TABLE 1:** Primer sequences used for qPCR assays

Gene	Forward	Reverse	Vendor
RPLP0	CACCATTGAAATCCTGAGTGATGT	TGACCAGCCCAAAGGAGAAG	IDT
FLG	GAGCTGAAGGAACTTCTGG	GATCCATGAAGACATCAACCA	Invitrogen, IDT
LOR	ACCCTTCCTGGTGCTTTG	CAGAGGTCTTCACGCAGTC	IDT
TGM1	AGTTCACAGTCCGCACAC	TTAAGAACATACTCCTGCCGC	IDT
HPRT	ACAGAGGGCTACAATGTGATG	TGCTGAGGATTTGGAAAGGG	IDT
IL-6	CTCTTCAGAACGAATTGAC	CTGCCAGTGCCTCTTT	Sigma
IL-8	AAACCACCGGAAGGAACCAT	GCTGCAGAAATCAGGAAGGC	Sigma
TNF	AGGCAGTCAGATCATCTTCTCG	TCTTGATGGCAGAGAGGAGG	Invitrogen

### Flow cytometry

Cells were plated at a density of 300,000 cells/well in a 12-well plate. Two days later, cells were trypsinized with TrypLE (ThermoFisher Scientific), neutralized with DMEM containing 10% fetal bovine serum and centrifuged at 1500 RPM for 5 min. Cells were resuspended in FACS buffer (phosphate-buffered saline containing 1 mM EDTA, 3% fetal bovine serum [FBS]) and counted. Cells were centrifuged at 1500 RPM for 5 min and washed twice with FACS buffer. Cells were resuspended in human Trustain FCX (5 µL/1E6 cells) in 50 µL FACS buffer. Cells were incubated on ice for 20 min. Then, 50 µL of the primary antibody cocktail [1:100 dilution of anti-CD40 (RD Clone 8211 catalog # MAB6321) in FACS buffer] was added and cells were incubated on ice for 1 h. Cells were centrifuged at 1500 RPM for 5 min and resuspended in 50 µL of the secondary antibody cocktail [1:500 dilution of anti-Mouse IgG Alexa fluo488 (Invitrogen) in FACS buffer]. Cells were incubated on ice for 1 h. Cells were centrifuged at 1500 RPM for 5 min, washed with FACS buffer three times, and then resuspended in 150 µL of FACS buffer. Stained cells were analyzed using the Accuri C6. Representative plots were made in FlowJo.

### CRISPR/Cas9 editing of keratinocytes

A CRISPR/Cas9 knockout kit targeting *CD40* (Gene Knockout Kit v2) was obtained through Synthego. The kit consisted of recombinant Cas9 protein and three sgRNAs with the following sequences: (i) AAUCUGUUGACCCCAAGC, (ii) AGGCUGGCACUGUACGAGUG, and (iii) UCUUCUCAGACCUAGGGCUU. sgRNAs and Cas9 protein (RNP mixture) were prepared as per Synthego’s instructions. For electroporation, the Neon Transfection system (ThermoFisher Scientific) was filled with 3 mL of electrolytic buffer and the following settings were used: voltage = 1400, width = 20, pulses = 2. Cells were trypsinized with TrypLE, neutralized with DMEM containing 10% FBS, and centrifuged at 1250 revolutions/min (RPM) for 5 min. Approximately 5 × 10^5^ cells were pelleted, and the conditioned medium was removed. Cells were then resuspended in 12 µL of 3:1 Resuspension Buffer R and premixed RNP. Reaction mix (10 µL) was used for electroporation. Electroporated cells were added to a 6-well plate containing prewarmed media and expanded.

### Monoclonal selection of CD40 KO cells

Polyclonal CD40 KO cells were trypsinized, centrifuged, and then resuspended in keratinocyte serum-free medium (KSFM). Approximately 500 cells were added to the uppermost left well of a 96-well plate. Serial twofold dilutions were performed down the first column of the plate, followed by twofold dilutions across all rows of the plate. Wells that contained a single cell were used for clonal selection, grown to ~30% confluency and expanded.

### DNA isolation, PCR, and gel electrophoresis

CD40 KO clones and polyclonal populations (10^6^ cells) were processed for genomic DNA isolation using the PureLink Genomic DNA Mini Kit (ThermoFisher Scientific). Isolated DNA was resuspended in nuclease-free water and quantified by UV-Vis absorbance using a Nanodrop lite Spectrophotometer (Thermo Scientific). Next, PCR was used to amplify the edited region of *CD40*. Each PCR contained 7 µL of nuclease-free water, 10 µL of Accustart (Quantabio), 2 µL of primer mix (Forward:CTGCCACCAGCACAAATACT, Reverse: GAATGAACAAGGTCCCGTCT) at 10 µM and 1 µL of gDNA at 10 ng/µL. PCR amplification for *CD40* was performed using a SimpliAmp Thermal Cycler (Thermo Fisher Scientific) with the following protocol: 95°C for 2 min, 40 cycles (95°C for 20 s, 57°C for 30 s, and 72°C for 30 s), 72°C for 2 min, and at 4°C. After PCR was performed, 10 µL of the product was added to a 2.5% agarose gel containing GelRed Nucleic Acid Gel Stain (Biotium) and electrophoresed for 1 h at 120 volts. Gels were imaged using a BioRad Gel Imaging System, and the bands of interest were excised. DNA was extracted from the agar using a QIAquick Gel Extraction Kit (Qiagen). Isolated DNA was sequenced (Genewiz) using the same primers from the amplification step.

## Data Availability

No large datasets were generated or utilized during the current study.

## References

[1] Silverberg JI , Barbarot S , Gadkari A , Simpson EL , Weidinger S , Mina-Osorio P , Rossi AB , Brignoli L , Saba G , Guillemin I , Fenton MC , Auziere S , Eckert L . 2021. Atopic dermatitis in the pediatric population: a cross-sectional, international epidemiologic study. Ann Allergy Asthma Immunol 126:417–428. doi:10.1016/j.anai.2020.12.020 33421555

[2] Chiesa Fuxench ZC , Block JK , Boguniewicz M , Boyle J , Fonacier L , Gelfand JM , Grayson MH , Margolis DJ , Mitchell L , Silverberg JI , Schwartz L , Simpson EL , Ong PY . 2019. Atopic dermatitis in America study: a cross-sectional study examining the prevalence and disease burden of atopic dermatitis in the US adult population. J Invest Dermatol 139:583–590. doi:10.1016/j.jid.2018.08.028 30389491

[3] Weidinger S. , Novak N . 2016. Atopic dermatitis. Lancet 387:1109–1122. doi:10.1016/S0140-6736(15)00149-X 26377142

[4] Bantz SK , Zhu Z , Zheng T . 2014. The atopic march: progression from atopic dermatitis to allergic rhinitis and asthma. J Clin Cell Immunol 5:202. doi:10.4172/2155-9899.1000202 25419479 PMC4240310

[5] Weidinger Stephan , Beck LA , Bieber T , Kabashima K , Irvine AD . 2018. Atopic dermatitis. Nat Rev Dis Primers 4:1. doi:10.1038/s41572-018-0001-z 29930242

[6] Flohr C , England K , Radulovic S , McLean WHI , Campbel LE , Barker J , Perkin M , Lack G . 2010. Filaggrin loss-of-function mutations are associated with early-onset eczema, eczema severity and transepidermal water loss at 3 months of age. Br J Dermatol 163:1333–1336. doi:10.1111/j.1365-2133.2010.10068.x 21137118

[7] Gao P-S , Rafaels NM , Hand T , Murray T , Boguniewicz M , Hata T , Schneider L , Hanifin JM , Gallo RL , Gao L , Beaty TH , Beck LA , Barnes KC , Leung DYM . 2009. Filaggrin mutations that confer risk of atopic dermatitis confer greater risk for eczema herpeticum. J Allergy Clin Immunol 124:507–513, doi:10.1016/j.jaci.2009.07.034 19733298 PMC5103856

[8] Halling-Overgaard A-S , Kezic S , Jakasa I , Engebretsen KA , Maibach H , Thyssen JP . 2017. Skin absorption through atopic dermatitis skin: a systematic review. Br J Dermatol 177:84–106. doi:10.1111/bjd.15065 27639188

[9] Miajlovic H , Fallon PG , Irvine AD , Foster TJ . 2010. Effect of filaggrin breakdown products on growth of and protein expression by Staphylococcus aureus. J Allergy Clin Immunol 126:1184–90. doi:10.1016/j.jaci.2010.09.015 21036388 PMC3627960

[10] Kong HH , Oh J , Deming C , Conlan S , Grice EA , Beatson MA , Nomicos E , Polley EC , Komarow HD , NISC Comparative Sequence Program, Murray PR , Turner ML , Segre JA . 2012. Temporal shifts in the skin microbiome associated with disease flares and treatment in children with atopic dermatitis. Genome Res 22:850–859. doi:10.1101/gr.131029.111 22310478 PMC3337431

[11] Tauber M , Balica S , Hsu C-Y , Jean-Decoster C , Lauze C , Redoules D , Viodé C , Schmitt A-M , Serre G , Simon M , Paul CF . 2016. Staphylococcus aureus density on lesional and nonlesional skin is strongly associated with disease severity in atopic dermatitis. J Allergy Clin Immunol 137:1272–1274. doi:10.1016/j.jaci.2015.07.052 26559326

[12] Totté JEE , van der Feltz WT , Hennekam M , van Belkum A , van Zuuren EJ , Pasmans SGMA . 2016. Prevalence and odds of Staphylococcus aureus carriage in atopic dermatitis: a systematic review and meta-analysis. Br J Dermatol 175:687–695. doi:10.1111/bjd.14566 26994362

[13] Simpson EL , Villarreal M , Jepson B , Rafaels N , David G , Hanifin J , Taylor P , Boguniewicz M , Yoshida T , De Benedetto A , Barnes KC , Leung DYM , Beck LA . 2018. Patients with atopic dermatitis colonized with Staphylococcus aureus have a distinct phenotype and endotype. J Invest Dermatol 138:2224–2233. doi:10.1016/j.jid.2018.03.1517 29604251 PMC6153055

[14] Deckers IAG , McLean S , Linssen S , Mommers M , van Schayck CP , Sheikh A . 2012. Investigating international time trends in the incidence and prevalence of atopic eczema 1990-2010: a systematic review of epidemiological studies. PLoS One 7:e39803. doi:10.1371/journal.pone.0039803 22808063 PMC3394782

[15] Meylan P , Lang C , Mermoud S , Johannsen A , Norrenberg S , Hohl D , Vial Y , Prod’hom G , Greub G , Kypriotou M , Christen-Zaech S . 2017. Skin colonization by Staphylococcus aureus precedes the clinical diagnosis of atopic dermatitis in infancy. J Invest Dermatol 137:2497–2504. doi:10.1016/j.jid.2017.07.834 28842320

[16] Werner Y , Lindberg M . 1985. Transepidermal water loss in dry and clinically normal skin in patients with atopic dermatitis. Acta Derm Venereol 65:102–105. 2408409

[17] Kim BE , Leung DYM . 2018. Significance of skin barrier dysfunction in atopic dermatitis. Allergy Asthma Immunol Res 10:207–215. doi:10.4168/aair.2018.10.3.207 29676067 PMC5911439

[18] Chamlin SL , Kao J , Frieden IJ , Sheu MY , Fowler AJ , Fluhr JW , Williams ML , Elias PM . 2002. Ceramide-dominant barrier repair lipids alleviate childhood atopic dermatitis: changes in barrier function provide a sensitive indicator of disease activity. J Am Acad Dermatol 47:198–208. doi:10.1067/mjd.2002.124617 12140465

[19] De Benedetto A , Rafaels NM , McGirt LY , Ivanov AI , Georas SN , Cheadle C , Berger AE , Zhang K , Vidyasagar S , Yoshida T , Boguniewicz M , Hata T , Schneider LC , Hanifin JM , Gallo RL , Novak N , Weidinger S , Beaty TH , Leung DYM , Barnes KC , Beck LA . 2011. Tight junction defects in patients with atopic dermatitis. J Allergy Clin Immunol 127:773–86. doi:10.1016/j.jaci.2010.10.018 21163515 PMC3049863

[20] De Benedetto A , Slifka MK , Rafaels NM , Kuo I-H , Georas SN , Boguniewicz M , Hata T , Schneider LC , Hanifin JM , Gallo RL , Johnson DC , Barnes KC , Leung DYM , Beck LA . 2011. Reductions in claudin-1 may enhance susceptibility to herpes simplex virus 1 infections in atopic dermatitis. J Allergy Clin Immunol 128:242–246. doi:10.1016/j.jaci.2011.02.014 21489616 PMC3129463

[21] Irvine AD , McLean WHI , Leung DYM . 2011. Filaggrin mutations associated with skin and allergic diseases. N Engl J Med 365:1315–1327. doi:10.1056/NEJMra1011040 21991953

[22] Berdyshev E , Goleva E , Bronova I , Dyjack N , Rios C , Jung J , Taylor P , Jeong M , Hall CF , Richers BN , Norquest KA , Zheng T , Seibold MA , Leung DY . 2018. Lipid abnormalities in atopic skin are driven by type 2 cytokines. JCI Insight 3:e98006. doi:10.1172/jci.insight.98006 29467325 PMC5916244

[23] Pellerin L , Henry J , Hsu C-Y , Balica S , Jean-Decoster C , Méchin M-C , Hansmann B , Rodriguez E , Weindinger S , Schmitt A-M , Serre G , Paul C , Simon M . 2013. Defects of filaggrin-like proteins in both lesional and nonlesional atopic skin. J Allergy Clin Immunol 131:1094–1102. doi:10.1016/j.jaci.2012.12.1566 23403047

[24] Igawa S , Kishibe M , Minami-Hori M , Honma M , Tsujimura H , Ishikawa J , Fujimura T , Murakami M , Ishida-Yamamoto A . 2017. Incomplete KLK7 secretion and upregulated LEKTI expression underlie hyperkeratotic stratum corneum in atopic dermatitis. J Invest Dermatol 137:449–456. doi:10.1016/j.jid.2016.10.015 27769847

[25] Rawlings AV , Voegeli R . 2013. Stratum corneum proteases and dry skin conditions. Cell Tissue Res 351:217–235. doi:10.1007/s00441-012-1501-x 23053051

[26] Ishikawa J , Narita H , Kondo N , Hotta M , Takagi Y , Masukawa Y , Kitahara T , Takema Y , Koyano S , Yamazaki S , Hatamochi A . 2010. Changes in the ceramide profile of atopic dermatitis patients. J Invest Dermatol 130:2511–2514. doi:10.1038/jid.2010.161 20574438

[27] Janssens M , van Smeden J , Gooris GS , Bras W , Portale G , Caspers PJ , Vreeken RJ , Hankemeier T , Kezic S , Wolterbeek R , Lavrijsen AP , Bouwstra JA . 2012. Increase in short-chain ceramides correlates with an altered lipid organization and decreased barrier function in atopic eczema patients. J Lipid Res 53:2755–2766. doi:10.1194/jlr.P030338 23024286 PMC3494247

[28] Yoshida K , Kubo A , Fujita H , Yokouchi M , Ishii K , Kawasaki H , Nomura T , Shimizu H , Kouyama K , Ebihara T , Nagao K , Amagai M . 2014. Distinct behavior of human Langerhans cells and inflammatory dendritic epidermal cells at tight junctions in patients with atopic dermatitis. J Allergy Clin Immunol 134:856–864. doi:10.1016/j.jaci.2014.08.001 25282566

[29] Danso M , Boiten W , van Drongelen V , Gmelig Meijling K , Gooris G , El Ghalbzouri A , Absalah S , Vreeken R , Kezic S , van Smeden J , Lavrijsen S , Bouwstra J . 2017. Altered expression of epidermal lipid bio-synthesis enzymes in atopic dermatitis skin is accompanied by changes in stratum corneum lipid composition. J Dermatol Sci 88:57–66. doi:10.1016/j.jdermsci.2017.05.005 28571749

[30] Di Nardo A , Wertz P , Giannetti A , Seidenari S . 1998. Ceramide and cholesterol composition of the skin of patients with atopic dermatitis. Acta Derm Venereol 78:27–30. doi:10.1080/00015559850135788 9498022

[31] van Smeden J , Bouwstra JA . 2016. Stratum Corneum lipids: their role for the skin barrier function in healthy subjects and atopic dermatitis patients. Curr Probl Dermatol 49:8–26. doi:10.1159/000441540 26844894

[32] Langan SM , Abuabara K , Henrickson SE , Hoffstad O , Margolis DJ . 2017. Increased risk of cutaneous and systemic infections in atopic dermatitis-A cohort study. J Invest Dermatol 137:1375–1377. doi:10.1016/j.jid.2017.01.030 28202403 PMC5660507

[33] Werfel T , Ballmer-Weber B , Eigenmann PA , Niggemann B , Rancé F , Turjanmaa K , Worm M . 2007. Eczematous reactions to food in atopic eczema: position paper of the EAACI and GA2LEN. Allergy 62:723–728. doi:10.1111/j.1398-9995.2007.01429.x 17573718

[34] Xia J , Huang CL , Chu P , Kroshinsky D . 2022. Eczema monkeypoxicum: report of monkeypox transmission in a patient with atopic dermatitis. JAAD Case Rep 29:95–99. doi:10.1016/j.jdcr.2022.08.034 36212897 PMC9534102

[35] Ong PY , Leung DYM . 2016. Bacterial and viral infections in atopic dermatitis: a comprehensive review. Clinic Rev Allerg Immunol 51:329–337. doi:10.1007/s12016-016-8548-5 27377298

[36] Beck LA , Boguniewicz M , Hata T , Schneider LC , Hanifin J , Gallo R , Paller AS , Lieff S , Reese J , Zaccaro D , Milgrom H , Barnes KC , Leung DYM . 2009. Phenotype of atopic dermatitis subjects with a history of eczema herpeticum. J Allergy Clin Immunol 124:260–269, doi:10.1016/j.jaci.2009.05.020 19541356 PMC3056058

[37] Schlievert PM . 2016. Edited by G. A. Somerville . Staphylococcus: genetics and physiology. Caister Academic Press.

[38] Williams MR , Nakatsuji T , Sanford JA , Vrbanac AF , Gallo RL . 2017. Staphylococcus aureus induces increased serine protease activity in keratinocytes. J Invest Dermatol 137:377–384. doi:10.1016/j.jid.2016.10.008 27765722 PMC5258850

[39] Ohnemus U , Kohrmeyer K , Houdek P , Rohde H , Wladykowski E , Vidal S , Horstkotte MA , Aepfelbacher M , Kirschner N , Behne MJ , Moll I , Brandner JM . 2008. Regulation of epidermal tight-junctions (TJ) during infection with exfoliative toxin-negative Staphylococcus strains. J Invest Dermatol 128:906–916. doi:10.1038/sj.jid.5701070 17914452

[40] Bin L , Kim BE , Brauweiler A , Goleva E , Streib J , Ji Y , Schlievert PM , Leung DYM . 2012. Staphylococcus aureus alpha-toxin modulates skin host response to viral infection. J Allergy Clin Immunol 130:683–691. doi:10.1016/j.jaci.2012.06.019 22840852 PMC3594992

[41] Schlievert PM , Roller RJ , Kilgore SH , Villarreal M , Klingelhutz AJ , Leung DYM . 2021. Staphylococcal TSST-1 association with eczema herpeticum in humans. mSphere 6:e0060821. doi:10.1128/mSphere.00608-21 34319127 PMC8386428

[42] Boguniewicz M , Leung DYM . 2010. Recent insights into atopic dermatitis and implications for management of infectious complications. J Allergy Clin Immunol 125:4–13; doi:10.1016/j.jaci.2009.11.027 20109729 PMC2814072

[43] Schlievert PM , Case LC , Strandberg KL , Abrams BB , Leung DYM . 2008. Superantigen profile of Staphylococcus aureus isolates from patients with steroid-resistant atopic dermatitis. Clin Infect Dis 46:1562–1567. doi:10.1086/586746 18419342 PMC2637450

[44] Tuffs SW , Haeryfar SMM , McCormick JK . 2018. Manipulation of innate and adaptive immunity by Staphylococcal superantigens. Pathogens 7:53. doi:10.3390/pathogens7020053 29843476 PMC6027230

[45] King JM , Kulhankova K , Stach CS , Vu BG , Salgado-Pabón W . 2016. Phenotypes and virulence among Staphylococcus aureus USA200, USA300, USA400, and USA600 clonal lineages. mSphere 1:e00071-16. doi:10.1128/mSphere.00071-16 27303750 PMC4899884

[46] Herbert S , Ziebandt A-K , Ohlsen K , Schäfer T , Hecker M , Albrecht D , Novick R , Götz F . 2010. Repair of global regulators in Staphylococcus aureus 8325 and comparative analysis with other clinical isolates. Infect Immun 78:2877–2889. doi:10.1128/IAI.00088-10 20212089 PMC2876537

[47] Nair D , Memmi G , Hernandez D , Bard J , Beaume M , Gill S , Francois P , Cheung AL . 2011. Whole-genome sequencing of Staphylococcus aureus strain RN4220, a key laboratory strain used in virulence research, identifies mutations that affect not only virulence factors but also the fitness of the strain. J Bacteriol 193:2332–2335. doi:10.1128/JB.00027-11 21378186 PMC3133102

[48] Miller LG , Perdreau-Remington F , Rieg G , Mehdi S , Perlroth J , Bayer AS , Tang AW , Phung TO , Spellberg B . 2005. Necrotizing fasciitis caused by community-associated methicillin-resistant Staphylococcus aureus in Los Angeles. N Engl J Med 352:1445–1453. doi:10.1056/NEJMoa042683 15814880

[49] Schlievert PM , Strandberg KL , Lin Y-C , Peterson ML , Leung DYM . 2010. Secreted virulence factor comparison between methicillin-resistant and methicillin-sensitive Staphylococcus aureus, and its relevance to atopic dermatitis. J Allergy Clin Immunol 125:39–49. doi:10.1016/j.jaci.2009.10.039 20109735 PMC2814367

[50] King MD , Humphrey BJ , Wang YF , Kourbatova EV , Ray SM , Blumberg HM . 2006. Emergence of community-acquired methicillin-resistant Staphylococcus aureus USA 300 clone as the predominant cause of skin and soft-tissue infections. Ann Intern Med 144:309–317. doi:10.7326/0003-4819-144-5-200603070-00005 16520471

[51] Bonnstetter KK , Wolter DJ , Tenover FC , McDougal LK , Goering RV . 2007. Rapid multiplex PCR assay for identification of USA300 community-associated methicillin-resistant Staphylococcus aureus isolates. J Clin Microbiol 45:141–146. doi:10.1128/JCM.01228-06 17093011 PMC1828973

[52] Yeung M , Balma-Mena A , Shear N , Simor A , Pope E , Walsh S , McGavin MJ . 2011. Identification of major clonal complexes and toxin producing strains among Staphylococcus aureus associated with atopic dermatitis. Microbes Infect 13:189–197. doi:10.1016/j.micinf.2010.10.023 21093604

[53] Brewer MG , Monticelli SR , Moran MC , Miller BL , Beck LA , Ward BM . 2022. Conditions that simulate the environment of atopic dermatitis enhance susceptibility of human keratinocytes to vaccinia virus. Cells 11:1337. doi:10.3390/cells11081337 35456017 PMC9025056

[54] Moran MC , Pandya RP , Leffler KA , Yoshida T , Beck LA , Brewer MG . 2021. Characterization of human keratinocyte cell lines for barrier studies. JID Innov 1:100018. doi:10.1016/j.xjidi.2021.100018 34909717 PMC8659750

[55] Moran MC , Chinchilli E , Kenney HM , Pope EM , Scott G , Brewer MG , Beck LA . 2023. Stage of keratinocyte differentiation is a key determinant of viral susceptibility in human skin. J Invest Dermatol:S0022-202X(23)01937-1. doi:10.1016/j.jid.2023.03.1656 PMC10524096 36997109

[56] Moran MC , Cahill MP , Brewer MG , Yoshida T , Knowlden S , Perez-Nazario N , Schlievert PM , Beck LA . 2019. Staphylococcal virulence factors on the skin of atopic dermatitis patients. mSphere 4:e00616-19. doi:10.1128/mSphere.00616-19 31826969 PMC6908420

[57] Proft T , Fraser JD . 2003. Bacterial superantigens. Clin Exp Immunol 133:299–306. doi:10.1046/j.1365-2249.2003.02203.x 12930353 PMC1808794

[58] Messingham KN , Cahill MP , Kilgore SH , Munjal A , Schlievert PM , Fairley JA . 2022. TSST-1(+)Staphylococcus aureus in bullous pemphigoid. J Invest Dermatol 142:1032–1039. doi:10.1016/j.jid.2021.08.438 34606884 PMC9215375

[59] Schlievert PM , Peterson ML , Kaufmann GF . 2012. Glycerol monolaurate antibacterial activity in broth and biofilm cultures. PLoS ONE 7:e40350. doi:10.1371/journal.pone.0040350 22808139 PMC3394780

[60] Goleva E , Berdyshev E , Leung DY . 2019. Epithelial barrier repair and prevention of allergy. J Clin Invest 129:1463–1474. doi:10.1172/JCI124608 30776025 PMC6436854

[61] Schlievert PM , Cahill MP , Hostager BS , Brosnahan AJ , Klingelhutz AJ , Gourronc FA , Bishop GA , Leung DYM . 2019. Staphylococcal superantigens stimulate epithelial cells through CD40 to produce chemokines. mBio 10:e00214-19. doi:10.1128/mBio.00214-19 30890614 PMC6426597

[62] Denfeld RW , Hollenbaugh D , Fehrenbach A , Weiss JM , von Leoprechting A , Mai B , Voith U , Schöpf E , Aruffo A , Simon JC . 1996. CD40 is functionally expressed on human keratinocytes. Eur J Immunol 26:2329–2334. doi:10.1002/eji.1830261009 8898941

[63] Péguet-Navarro J , Dalbiez-Gauthier C , Moulon C , Berthier O , Réano A , Gaucherand M , Banchereau J , Rousset F , Schmitt D . 1997. Cd40 ligation of human Keratinocytes inhibits their proliferation and induces their differentiation. J Immunol 158:144–152. doi:10.4049/jimmunol.158.1.144 8977185

[64] Companjen AR , van der Wel LI , Boon L , Prens EP , Laman JD . 2002. CD40 ligation-induced cytokine production in human skin explants is partly mediated via IL-1. Int Immunol 14:669–676. doi:10.1093/intimm/dxf033 12039918

[65] Brewer MG , Anderson EA , Pandya RP , De Benedetto A , Yoshida T , Hilimire TA , Martinez-Sobrido L , Beck LA , Miller BL . 2020. Peptides derived from the tight junction protein CLDN1 disrupt the skin barrier and promote responsiveness to an epicutaneous vaccine. J Invest Dermatol 140:361–369. doi:10.1016/j.jid.2019.06.145 31381894

[66] Nakatsuji T , Chiang H-I , Jiang SB , Nagarajan H , Zengler K , Gallo RL . 2013. The microbiome extends to subepidermal compartments of normal skin. Nat Commun 4:1431. doi:10.1038/ncomms2441 23385576 PMC3655727

[67] Nakatsuji T , Chen TH , Two AM , Chun KA , Narala S , Geha RS , Hata TR , Gallo RL . 2016. Staphylococcus aureus exploits epidermal barrier defects in atopic dermatitis to trigger cytokine expression. J Invest Dermatol 136:2192–2200. doi:10.1016/j.jid.2016.05.127 27381887 PMC5103312

[68] Bäsler K , Galliano M-F , Bergmann S , Rohde H , Wladykowski E , Vidal-Y-Sy S , Guiraud B , Houdek P , Schüring G , Volksdorf T , Caruana A , Bessou-Touya S , Schneider SW , Duplan H , Brandner JM . 2017. Biphasic influence of Staphylococcus aureus on human epidermal tight junctions. Ann N Y Acad Sci 1405:53–70. doi:10.1111/nyas.13418 28753223

[69] Eckert RL , Crish JF , Robinson NA . 1997. The epidermal keratinocyte as a model for the study of gene regulation and cell differentiation. Physiol Rev 77:397–424. doi:10.1152/physrev.1997.77.2.397 9114819

[70] Bin L , Howell MD , Kim BE , Streib JE , Hall CF , Leung DYM . 2011. Specificity protein 1 is pivotal in the skin’s antiviral response. J Allergy Clin Immunol 127:430–438. doi:10.1016/j.jaci.2010.11.013 21208652 PMC3734863

[71] Takagi A , Nishiyama C , Maeda K , Tokura T , Kawada H , Kanada S , Niwa Y , Nakano N , Mayuzumi N , Nishiyama M , Ikeda S , Okumura K , Ogawa H . 2008. Role of Sp1 in transcription of human ATP2A2 gene in Keratinocytes. J Invest Dermatol 128:96–103. doi:10.1038/sj.jid.5700937 17597815

[72] Kawada H , Nishiyama C , Takagi A , Tokura T , Nakano N , Maeda K , Mayuzumi N , Ikeda S , Okumura K , Ogawa H . 2005. Transcriptional regulation of ATP2C1 gene by Sp1 and YY1 and reduced function of its promoter in Hailey-Hailey disease keratinocytes. J Invest Dermatol 124:1206–1214. doi:10.1111/j.0022-202X.2005.23748.x 15955096

[73] Howell MD , Gallo RL , Boguniewicz M , Jones JF , Wong C , Streib JE , Leung DYM . 2006. Cytokine milieu of atopic dermatitis skin subverts the innate immune response to vaccinia virus. Immunity 24:341–348. doi:10.1016/j.immuni.2006.02.006 16546102

[74] Howell MD , Jones JF , Kisich KO , Streib JE , Gallo RL , Leung DYM . 2004. Selective killing of vaccinia virus by LL-37: implications for eczema vaccinatum. J Immunol 172:1763–1767. doi:10.4049/jimmunol.172.3.1763 14734759

[75] Ong PY , Ohtake T , Brandt C , Strickland I , Boguniewicz M , Ganz T , Gallo RL , Leung DYM . 2002. Endogenous antimicrobial peptides and skin infections in atopic dermatitis. N Engl J Med 347:1151–1160. doi:10.1056/NEJMoa021481 12374875

[76] Banke E , Rödström K , Ekelund M , Dalla-Riva J , Lagerstedt JO , Nilsson S , Degerman E , Lindkvist-Petersson K , Nilson B . 2014. Superantigen activates the gp130 receptor on adipocytes resulting in altered adipocyte metabolism. Metabolism 63:831–840. doi:10.1016/j.metabol.2014.03.004 24684823

[77] Grousson J , Ffrench M , Concha M , Schmitt D , Péguet-Navarro J . 2000. CD40 ligation alters the cell cycle of differentiating keratinocytes. J Invest Dermatol 114:581–586. doi:10.1046/j.1523-1747.2000.00905.x 10692121

[78] Merriman JA , Mueller EA , Cahill MP , Beck LA , Paller AS , Hanifin JM , Ong PY , Schneider L , Babineau DC , David G , Lockhart A , Artis K , Leung DYM , Schlievert PM . 2016. Temporal and racial differences associated with atopic dermatitis Staphylococcus aureus and encoded virulence factors. mSphere 1:e00295-16. doi:10.1128/mSphere.00295-16 27981233 PMC5143412

[79] Fischer AJ , Kilgore SH , Singh SB , Allen PD , Hansen AR , Limoli DH , Schlievert PM . 2019. High prevalence of Staphylococcus aureus enterotoxin gene cluster superantigens in cystic fibrosis clinical isolates. Genes (Basel) 10:12. doi:10.3390/genes10121036 PMC6947208 31842331

[80] Lindsay JA . 2010. Genomic variation and evolution of Staphylococcus aureus. Int J Med Microbiol 300:98–103. doi:10.1016/j.ijmm.2009.08.013 19811948

[81] McCormick JK , Yarwood JM , Schlievert PM . 2001. Toxic shock syndrome and bacterial superantigens: an update. Annu Rev Microbiol 55:77–104. doi:10.1146/annurev.micro.55.1.77 11544350

[82] Spaulding AR , Salgado-Pabón W , Kohler PL , Horswill AR , Leung DYM , Schlievert PM . 2013. Staphylococcal and Streptococcal superantigen exotoxins. Clin Microbiol Rev 26:422–447. doi:10.1128/CMR.00104-12 23824366 PMC3719495

[83] Mitchell DT , Levitt DG , Schlievert PM , Ohlendorf DH . 2000. Structural evidence for the evolution of pyrogenic toxin superantigens. J Mol Evol 51:520–531. doi:10.1007/s002390010116 11116326

[84] Poumay Y , Roland IH , Leclercq-Smekens M , Leloup R . 1994. Basal detachment of the epidermis using dispase: tissue spatial organization and fate of integrin alpha 6 beta 4 and hemidesmosomes. J Invest Dermatol 102:111–117. doi:10.1111/1523-1747.ep12371742 8288902

[85] Dickson MA , Hahn WC , Ino Y , Ronfard V , Wu JY , Weinberg RA , Louis DN , Li FP , Rheinwald JG . 2000. Human keratinocytes that express hTERT and also bypass a P16(INK4A)-enforced mechanism that limits life span become immortal yet retain normal growth and differentiation characteristics. Mol Cell Biol 20:1436–1447. doi:10.1128/MCB.20.4.1436-1447.2000 10648628 PMC85304

[86] Smits JPH , Niehues H , Rikken G , van Vlijmen-Willems IMJJ , van de Zande GWHJF , Zeeuwen PLJM , Schalkwijk J , van den Bogaard EH . 2017. Immortalized N/TERT keratinocytes as an alternative cell source in 3D human epidermal models. Sci Rep 7:11838. doi:10.1038/s41598-017-12041-y 28928444 PMC5605545

